# Rehabilitative Approach Toward a Japanese Encephalitis Patient via Therapy Ball: A Case Report

**DOI:** 10.7759/cureus.30883

**Published:** 2022-10-30

**Authors:** Ketki Kunjarkar, Pallavi Harjpal, Snehal Samal

**Affiliations:** 1 Physiotherapy, Ravi Nair Physiotherapy College, Datta Meghe Institute of Medical Sciences, Wardha, IND; 2 Neurophysiotherapy, Ravi Nair Physiotherapy College, Datta Meghe Institute of Medical Sciences, Wardha, IND

**Keywords:** encephalitis, physiotherapy, therapy ball, rehabilitation, japanese encephalitis

## Abstract

The most widespread and dangerous mosquito-borne viral encephalitis is called Japanese encephalitis (JE). In India, the disease still mostly affects children. Japanese encephalitis virus (JEV) attacks the central nervous system and causes fever, headache, vomiting, meningeal inflammation, and altered consciousness. Therefore, the focus of JE treatment is on symptom management, and thorough supportive care is crucial to prevent neurological sequelae. In the case study reported here, a five-year-old girl complained of loss of deglutition, loss of speech, diminished tone, and developmental milestone losses. Management was planned for the child using a rehabilitative approach that exclusively includes the use of a therapy ball along with standard physiotherapy protocol. Objectives created indicated that both preventing complications and facilitating recovery were important. The treatment protocol was provided for a period of four weeks. Modified Ashworth scale (MAS), manual muscle testing (MMT), and gross motor function measure (GMFM-88) were assessed pre- and post-treatment. This case report mentions the importance of the effectiveness of therapy ball in rehabilitation in patients with JE.

## Introduction

The inflammation of brain tissue is known as encephalitis [1]. It could arise from a direct infection or through a hematogenous pathway [2]. Viruses like herpes simplex, Japanese B encephalitis, mumps virus, etc. are commonly responsible for encephalitis [3]. Another manifestation of JE is acute encephalitis syndrome (AES). It should be noted that JE only accounts for 10%-15% of instances of AES, whose etiology is mostly unknown. Also, JE is highly lethal in children and is spread by the Culex type of mosquitoes [4]. It is caused by the Japanese encephalitis virus (JEV). Since the early 1870s, encephalitis epidemics have been recorded in Japan, initially termed type B encephalitis. With an estimated 50,000 cases and 15,000 fatalities per year, JE is one of the most common viral encephalitis types in the world [5]. Later, it was discovered that the virus belonged to the family *Flaviviridae*, a genus named after the prototype yellow fever virus (flavi in Latin meaning yellow) [5].

In Asia, the most frequent cause of viral encephalitis is the JEV. Other names for JE include Japanese encephalitis (JE), Japanese brain fever, Arbovirus B, and Russian autumnal encephalitis. The incubation phase for JE lasts for four to 14 days. The majority of JEV infections have minimal symptoms, i.e., fever and headache. Gastrointestinal discomfort and vomiting may be the main early symptoms in children. Symptoms of a serious sickness include the sudden start of a high body temperature, headache, stiff neck, confusion, convulsions, spastic paralysis, and eventually death [6].

In India, epidemics of JE have been documented in numerous locations since 1955, making it one of the most serious problems affecting children's health with case-fatality rates ranging from 0.3% to 60%. Inactivated mouse brain-derived JE vaccine is available against JE in India [7]. Currently, the virus has a well-established zoonotic transmission cycle that involves pigs, aquatic birds, and/or mosquitoes; humans only get infected by accident when bitten by an infected mosquito and act as a dead-end host [8]. Infection can be avoided by limiting outdoor activity at night and dawn, wearing clothes that reveal the minimum amount of skin, getting the JE vaccine, and using mosquito repellent [5].

The purpose of treatment is to stabilize the patient and relieve symptoms [6]. However, neurological sequelae can be seen in 30%-50% of patients with JE [4]. Neurological sequelae like altered consciousness, weakness (monoparesis, hemiparesis, and quadriparesis), focal or generalized abnormal limb tone (hypertonia and hypotonia), focal or generalized abnormal limb reflexes (hyperreflexia and hyporeflexia), diagnosis of new-onset or recurrent seizures, or new or recurrent extrapyramidal movement disorders were noted [9]. Convulsions and high intracranial pressure are managed as part of the supportive care given to patients with JE.

The trunk and pelvic muscles are a part of the core. Those muscles are extremely essential for preserving the stability of the spine and pelvis [10]. To be precise, the muscles that make up the core are the front abdominal muscles, paraspinal muscles, back gluteal muscles that serve as the roof, hip girdle muscles, and pelvic girdle muscles that serve as the bottom musculature [11]. Pelvic stability exercises are required to work the lower trunk and hip muscles concurrently. The muscles aid in achieving correct postures for the lumbar and pelvic areas by supplying core stability [12]. Physio balls aid in trunk muscle activity, which results in healthy trunk muscular movement [13]. Many physiotherapists use a physio ball or another special treatment tool to help patients with their balance and trunk stability. When the exercises are executed on a physio ball rather than done on a plinth, the trunk muscles may be activated more effectively as a patient's posture is perturbed when a ball moves beneath them, and the muscles respond in order to help the patient maintain the ideal posture [14]. Due to the clinical symptoms found in JE, there is an impairment in activities of daily living, which further affects the quality of life of the individual. So far, physical therapy has been beneficial. It is necessary to preserve the airway, breathing, and circulation [15].

## Case presentation

Patient information

As narrated by parents, the five-year-old female patient was apparently fine one month back. Then, she suddenly develops acute pyrexia, which was relieved by consultation and medication given by their family doctor. The episodes of sudden fever continued two to three times monthly. But later, the child experienced a high-grade fever; the parents immediately rushed her to a nearby government hospital, where she was admitted to the general ward for two days. On her second day, she experienced her first episode of convulsions and was referred to a private hospital in their local place, and she was urgently admitted to the intensive care unit. Investigations like MRI was done, and the child was diagnosed with JE. During the period of stay in the ICU, the child developed a few episodes of convulsions for which medications were given and brought under control. It further leads to loss of speech, bilateral upper limb weakness, and left lower limb weakness. After a stay of six days in the ICU, the child was shifted to the general ward. As the child's condition was deteriorating and there was a lack of availability for physiotherapy treatment, the child was referred to Acharya Vinoba Bhave Rural Hospital for further treatment. The child was admitted to the hospital on the 29th of August 2022 with a complaint of high-grade fever, generalized weakness, altered speech, lack of deglutition, and loss of bowel and bladder movements. The child was referred for physiotherapy for further management on the 5th of September 2022.

Clinical findings

MRI Impression

**Figure 1:**
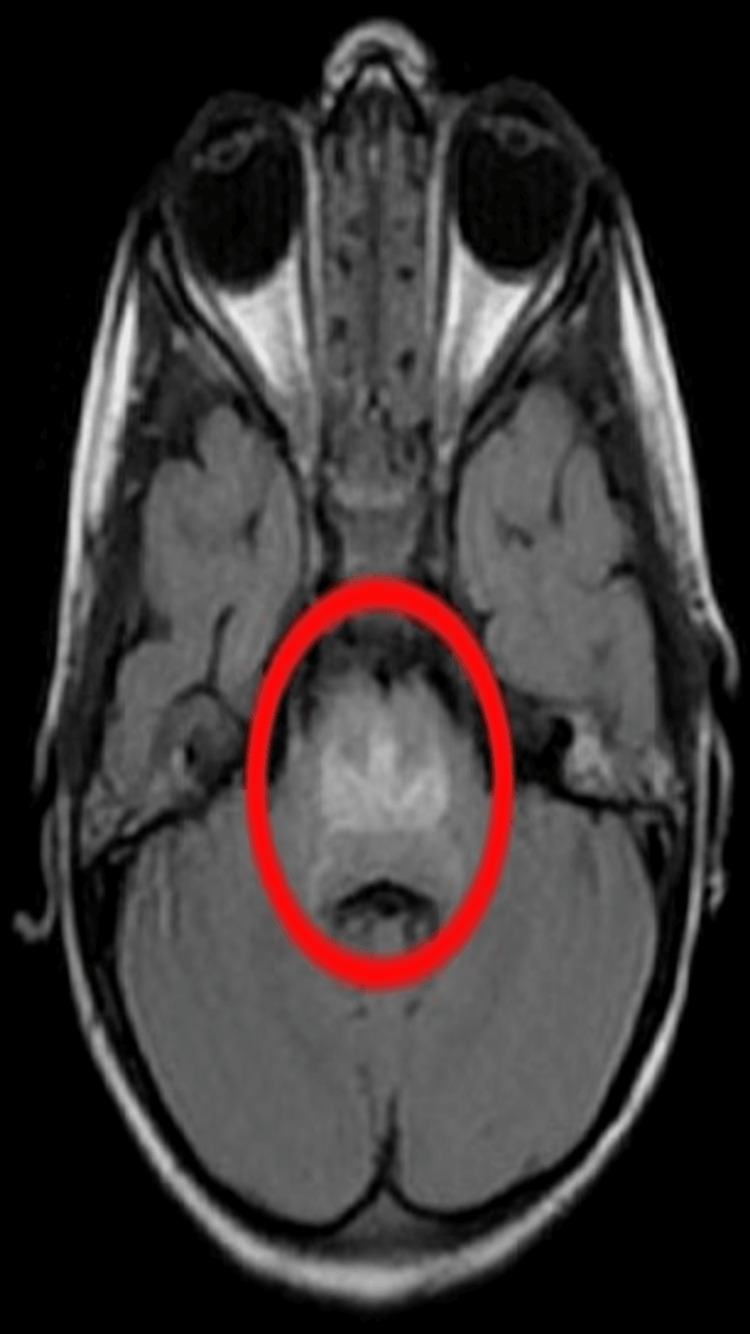
MRI of the brain (the focal lesions in the pons are indicated in the red circle)

Magnetic resonance image (MRI) report shows focal lesions in the pons depicted in Figure 1.

Physiotherapy assessment

The patient was conscious, cooperative, and well-oriented to time, person, and place. His hearing and vision were intact, except for his speech which was altered. Her body was afebrile, with heart rate of 80 beats per minute, respiratory rate of 22 cycles per minute, and blood pressure of 110/70 mmHg. The child had normal anthropometry measurements as per age. On observation, the child seems to be malnourished, the build was ectomorphic, and the skin appeared normal. There was a reduction in facial movements, and the lips were dry. With an attitude of the limb, the patient was seen in a supine line position with the ankle in slight plantar flexion, and the hip was externally rotated. During a neurological examination, sensations were intact. Initially, in both upper limbs and lower limbs, there was a reduction in tone. Later, after one week of intervention, there was a normalization in the tone. There was a gain in muscle strength with the right upper limb strength being grade 1 on manual muscle testing (MMT), and the right lower limb gained a strength of grade 2 on MMT. All the deep tendon reflexes were intact, except for the knee which was diminished on both sides. Babinski sign was positive. Also, there was the loss of developmental achieved milestones. The patient had a Ryle’s tube.

Physiotherapy intervention

**Figure 2:**
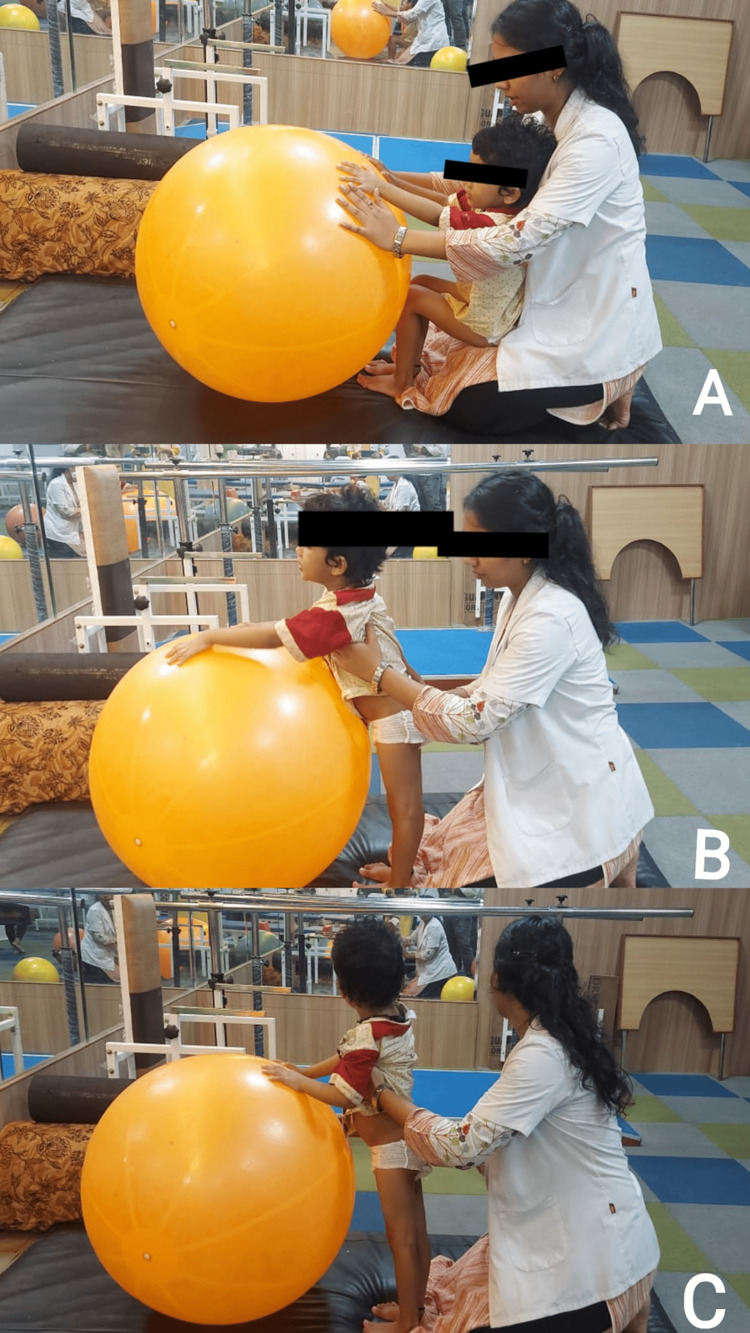
Sit-to-stand training for the patient to gain standing (A) The initial position for standing. (B) The patient tries to stand. (C) The patient is standing.

**Figure 3:**
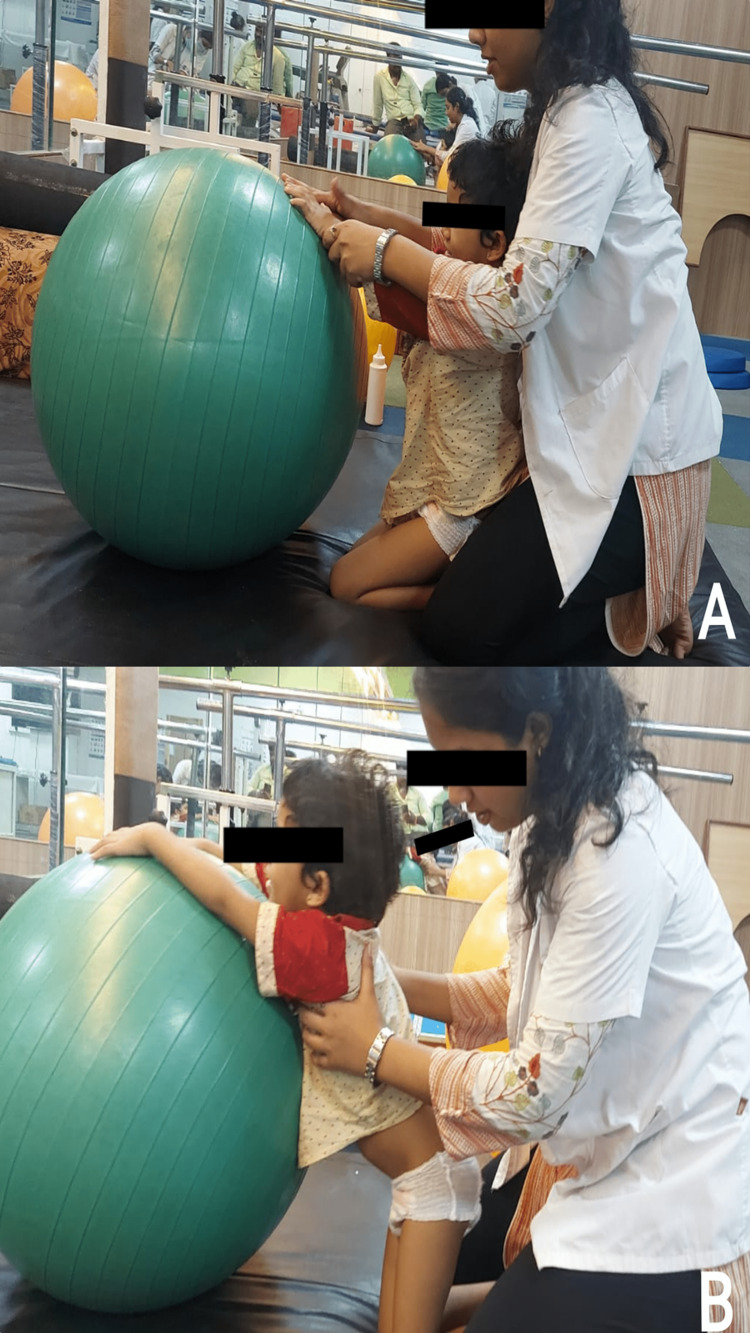
Kneel sitting to kneel standing position (required for transition training) (A) The patient in kneel sitting position and (B) the patient in kneel standing position.

The physiotherapy intervention protocol is depicted in Figures 2, 3, and Table 1.

**Table 1:** Physiotherapy intervention protocol for the patient in a span of four weeks

Problem identified	Goal	Treatment strategy	Intervention
Hypotonia	To develop tone in postural and phasic muscles	Facilitator’s techniques for postural and phasic muscles	Active assisted movements of the right lower limb for all joints (10 rep [repetition] x 1 set)
			Passive movements of left upper and lower limbs and right upper limb with rood’s tapping facilitation (10 rep x 1 set)
			Joint approximation (10 rep x 1 set)
			Upper limb strengthening with a 250-liter water bottle (10 rep x 1 set)
Lack of head and neck control	To enhance head and neck control	Facilitatory approaches and vestibular stimulation	Neck control facilitation techniques with bolsters (10 rep x 1 set)
			Head control in prone exercises with a wedge (10 rep x 1 set)
			Full body extension and supine lateral rolls with physio balls (10 rep x 1 set)
			Lap therapy (trunk rotations and reach-outs in sitting)
Lack of pelvic, trunk control and efficient rolling	To enhance pelvic, trunk control and promote segmental rolling	Rolling facilitation and mat activities	Weight shifting forward and backward movements in quadripod position and sideward weight shifting movements in cross-sitting position with back supported with bolsters
			Sitting on the physio ball (rocking the child forward, back, and side to side with pelvic support)
			Half kneeling, kneel sitting and standing, sit to stand with physio balls (Figures 2, 3)
			Upper limb proprioceptive neuromuscular facilitation (PNF) (D1 and D2 patterns)
			Pelvic PNF
			Pelvic bridging exercises (10 reps x 1 set)
			Weight-bearing on the elbow in side lying (10-sec hold x 2 sets)
			Half push-ups in cobra pose (5 reps x 2 sets)
Lack of speech and deglutition	To enhance speech and deglutition	Oro-motor rehabilitation and speech therapy	Oro-motor retraining
			Tongue range of motion (ROM) exercises
			Lip exercises
			Jaw exercises
			Consonant and vowel pairing
			Repetition
			Sentence production
			Phonological processing

Outcome measure

**Figure 4:**
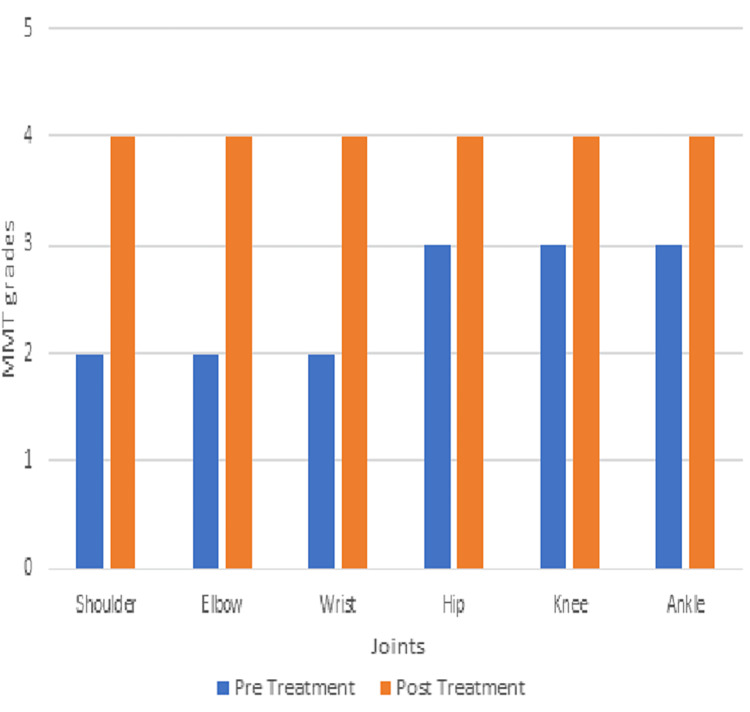
Manual muscle testing (MMT) for the right side

**Figure 5:**
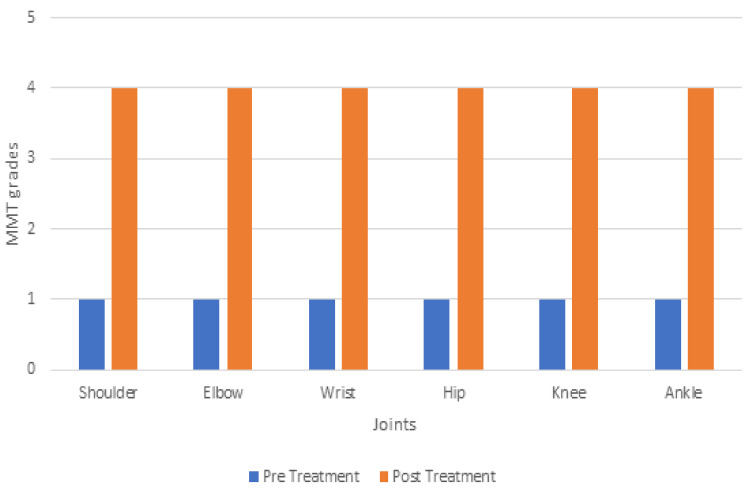
Manual muscle testing (MMT) for the left side

The MMT, gross motor function measure-88 (GMFM-88) [16], and modified Ashworth scale were tested pre- and post-intervention to measure the outcomes; this is indicated in Figures 4, 5, and Table 2.

**Table 2:** Gross motor function measure-88 (GMFM-88)

Component	Pre-treatment	Post-treatment
Lying and rolling	8	51
Sitting	0	43

## Discussion

The above case study intends to demonstrate how effective rehabilitation can be provided with the help of a therapy ball to a patient who has suffered from JE. This study's results are in line with those of a previous study conducted by Kale et al.; the duration of the physical therapy treatment was four weeks. Pre- and post-scores indicate that the child's tone and gross motor condition have both improved. The primary muscles of the upper and lower limbs were considered. Both sides of each of the three muscle groups showed an improvement in tone. According to the study, goal-oriented therapy is very important for raising a child's functional level [15].

The results of this study are consistent with those of Kale et al.'s study, in which the duration of the therapy was five months. Promising results can be shown with noticeable increases in muscle tone, stability, and several gait characteristics. The management of children with spastic paraplegic cerebral palsy via goal-directed therapy utilizing neurodevelopmental techniques (NDT), a task-oriented approach, and PNF has proven to be successful [17].

The results are also in line with a previous study, in which the authors examined the impact of goal-directed functional therapy (GDT) and activity-focused therapy (AT) on daily living skills and gross motor function in preschool-aged children with cerebral palsy (CP). In contrast to activity-oriented therapy, children with CP showed clear improvements in daily activities and gross motor function after receiving goal-driven, functional therapy [18]. The findings of the study create a crucial profile of patients with infectious encephalitis in a rehabilitation context, which can help with better planning and provision of rehabilitation treatments [19].

In this case, when the patient came for physiotherapy on her first day, she was unable to move both her upper limbs and left lower limb, had a lack of neck and head control, lack of speech, and deglutition. But when we started the treatment, the main aim was focused on bed mobility activities, transferring activities, and oro-motor retraining to make the patient perform her daily activities at ease, thereby helping to improve the quality of life of the patient. Additionally, the outcomes are consistent with the research where the swiss ball proved to be a useful tool for increasing trunk muscle activity, strengthening the core muscles of the trunk, maintaining stability in upright positions, and allowing freedom of movement for the upper limbs. As opposed to other adaptive aids, the swiss ball activates all weight-shifting kinds with fewer transitions, requiring less energy from the therapist. Compared to the traditional therapy strategy, this method is more efficient [20].

## Conclusions

However, the mortality for JE can range from 0.3% to 60%. Although there is no known treatment for JE, there are treatment measures that are quite successful. The main goals of treatment are to help the patient get over the illness and to reduce the severity of their clinical symptoms. In this case report, the patient received symptomatic and rehabilitative treatment, including medication, physical therapy with an exclusive approach via therapy ball, and speech therapy. With the help of the above treatment protocol, a drastic improvement in the patient’s health was seen. Lying, rolling, and sitting components were taken from GMFM-88 by keeping the condition of the child in mind. However, more high-quality studies are required to prove the value and significance of physiotherapy in treating JE patients because there is a dearth of physiotherapy care available in this population.
